# Effects of non-pharmacological interventions on sleep in patients with critical illness: a systematic review and network meta-analysis

**DOI:** 10.1038/s41598-026-39187-y

**Published:** 2026-02-09

**Authors:** Yutaka Matsuura, Etsuko Kita, Yukari Taneda, Mikiko Matsuda, Mika Kataoka, Masahiro Masuya, Keiko Fukuroku

**Affiliations:** Division of Nursing, Mie University Graduate School of Medicine, Tsu, Japan

**Keywords:** Intensive care, Network meta-analysis, Non-pharmacological interventions, Patients with critical illness, Sleep quality, Diseases, Health care, Medical research

## Abstract

Adequate sleep is vital for recovery in patients with critical illness, yet sleep in intensive care units (ICUs) is widely disrupted. Although various non-pharmacological strategies have been proposed, evidence remains inconsistent, and the most effective intervention for improving ICU sleep has not been clearly established. In this review, we evaluated the effectiveness of non-pharmacological interventions for sleep promotion and identified the most effective strategies for improving sleep quality among ICU patients. Studies implementing non-pharmacological sleep-promoting interventions and evaluating sleep quality in ICU patients were identified using MEDLINE and Cumulative Index to Nursing and Allied Health Literature. The protocol was registered in PROSPERO (CRD420251184156). Thirty-six studies encompassing 16 intervention types were included. A Bayesian network meta-analysis demonstrated significant improvements for aromatherapy, eye mask, eye mask and earplugs, music therapy, and multicomponent intervention compared with control groups. Surface under the cumulative ranking curves indicated that music therapy (94.9%) had the highest probability of being the most effective intervention, followed by aromatherapy (76.2%). These interventions may improve sleep by reducing environmental disturbances and stabilizing autonomic and neurotransmitter activity.

**Supplementary Information:**

The online version contains supplementary material available at 10.1038/s41598-026-39187-y.

## Introduction

Sleep consists of three to five 90-to-120-min sleep cycles that alternate between non-rapid eye movement (REM) and REM sleep. Non-REM sleep is divided into N1, N2, and N3 stages, with N3 also referred to as slow-wave sleep (SWS). Although sleep gradually deepens and reaches the N3 stage after approximately 30 min, this deep sleep can be disrupted by sleep disturbances and microarousal. Sleep functions include the recovery from fatigue and stress, maintenance of energy balance, modulation of neural circuits, and regulation of body temperature. In contrast, sleep disruptions can lead to reduced immunity^1^, impaired glucose tolerance, and increased sympathetic activity^2^. Therefore, ensuring adequate sleep plays a significant role in providing life support.

Patients admitted to the intensive care unit (ICU) undergo invasive treatments, such as surgery. Consequently, the levels of inflammatory cytokines (e.g., interleukin [IL]-6, IL-8, tumor necrosis factor-α), which regulate innate and adaptive immune responses, increase^3^. These inflammatory cytokines have sleep-inducing effects and may promote non-REM sleep, which is crucial for recovery from tissue injury and physiological stress resulting from invasive treatments or critical illness^4^. Therefore, ensuring adequate sleep is essential for patients with critical illness admitted to the ICU, particularly for maintaining physiological stability and homeostasis.

Nevertheless, sleep disruption is common among patients with critical illness due to multiple factors, including environmental stimuli (e.g., noise from conversations between medical staff or monitoring systems, light from medical equipment, and room lighting)^5,6^, physiological and pathophysiological conditions (e.g., pain, discomfort, anxiety, and circadian rhythm disruption)^7,8^, and nocturnal nursing care activities^9^.

Patients with critical illness in the ICU commonly experience sleep disturbances, characterized by sleep fragmentation, increased light sleep, and reduction in slow-wave sleep and REM sleep^10,11^. Sleep disruption in patients with critical illness is associated with delirium, increased pain, and ICU-acquired weakness^12–14^. Moreover, this condition often persists for several months after ICU discharge, indicating that sleep disruption may adversely affect long-term prognosis^15,16^. Therefore, maintaining sufficient sleep is crucial for patients with critical illness to recover from the disease and improve long-term outcomes.

Both pharmacological and non-pharmacological interventions have been implemented to improve sleep quality in the ICU. In terms of pharmacological intervention, clinical practice guidelines for the prevention and management of pain, agitation/sedation, delirium, immobility, and sleep disruption in adult patients in the ICU (the Pain, Agitation/Sedation, Delirium, Immobility, and Sleep Disruption [PADIS] guidelines) published by the Society of Critical Care Medicine recommend ramelteon, which is melatonin receptor agonist, administration is recommended over no melatonin to promote sleep in adult patients in the ICU^17^. A meta-analysis examining the effects of melatonin reported that melatonin administration in patients with critical illness improved the perceived sleep quality compared with no administration^18^. In contrast, non-pharmacological interventions, such as noise and light reduction, aromatherapy, and the use of eye masks, improve subjective or objective sleep quality among patients with critical illness^8,19,20^. In addition, multicomponent interventions that combine these are recommended to improve sleep quality according to the PADIS guidelines^17^. However, there are limitations, such as small sample size and variable utility of non-pharmacological interventions, across studies. Although a pairwise meta-analysis, in which the data of each study are statistically synthesized and analyzed, is performed to resolve this problem, traditional meta-analysis allows the comparison of only two groups (control vs. intervention group)^21,22^. As a result, direct comparison between intervention groups were not performed, and it is also not feasible to evaluate all of these interventions head-to-head. Consequently, it remains unclear which intervention is the most effective in improving sleep quality in the ICU.

Therefore, in this review, we aimed to evaluate the effectiveness of non-pharmacological interventions for sleep promotion and identify the most effective strategies for improving sleep among patients in the ICU by estimating relative effects through network meta-analysis.

## Methods

The protocol was registered in PROSPERO (CRD420251184156). This meta-analysis and systematic review was performed in accordance with the Preferred Reporting Items for Systematic reviews and Meta-Analyses (PRISMA) for Network Meta-Analysis guidelines^23^ (Supplementary Table S1).

### Search strategy and selection

To minimize bias, we conducted a literature search using two databases, MEDLINE and Cumulative Index to Nursing and Allied Health Literature, which are widely used in the medical field via EBSCO^24^. The databases were last accessed on October 20, 2025. The search terms included those related to intensive care (e.g., ICU, critically ill patients), non-pharmacological interventions (e.g., sleep care), and sleep (Table 1). However, Controlled vocabulary (e.g., MeSH terms) was not used because newly indexed articles may not yet have assigned subject headings, and terminology related to non-pharmacological sleep interventions varies across databases. Therefore, we employed a sensitive free-text search strategy rather than a controlled-vocabulary–based approach to maximize the retrieval of relevant studies, although we acknowledge that this may have reduced specificity.

**Table 1:** Search terms and strategies.

Unit
Intensive care unit OR ICU OR critical ill patient* OR acute patient* OR critically ill OR critical illness OR CCU OR coronary care unit OR MICU OR medical care unit OR SICU OR surgical care unit
Intervention
Nonpharmacol* OR non-pharmacol* OR non-pharmacological interventions OR nonpharmacological intervention OR sleep promot* OR sleep care OR earplug OR aromatherapy OR eye mask OR music OR relaxation OR environmental intervention OR physical therapy modalities OR mobile* OR cognitive behavioral therapy OR light therapy OR bright light therapy OR nurs* OR treatment OR therapy OR strateg*
Sleep
Sleep OR sleep quality OR sleep disturb* OR insomnia

Subsequently, four authors independently assessed eligibility and selected articles based on predefined criteria. In cases of disagreement regarding a study’s eligibility, the four authors resolved the discrepancies through discussion. The inclusion criteria were as follows: (1) studies involving patients admitted to the ICU aged ≥ 18 years; (2) randomized controlled trials (RCTs), controlled clinical trials (CCTs), controlled before and after studies (CBAs), or crossover trials; (3) studies that included non-pharmacological interventions to improve sleep; (4) studies that used reliable and validated self-reported measurements to evaluate sleep quality; (5) studies reporting adequate data for pooling in the analysis (e.g., mean and standard deviation); and (6) studies published in English. The exclusion criteria were as follows: (1) studies that were not conducted on patients in critical care, (2) studies on pediatric patients, (3) studies on pharmacological interventions, and (4) non-original studies, such as case reports and reviews. The database search covered the period from 1968 to 2025 and did not apply any filters. Details of search strategy are provided in Supplementary Table S2.

### Data extraction

After selecting the studies based on the eligibility criteria, the following data were extracted: title, name of the first author, year of publication, country, study design, population, type of ICU, sample size, comparable group, intervention, average age, and assessment tool used. Additionally, the mean and standard deviation of the sleep assessment scores were extracted to pool the data from each group.

### Quality assessment

Four reviewers independently assessed the risk of bias in the RCTs using the Cochrane Risk of Bias tool for randomized trials (RoB 2). RoB 2 comprises five domains and an overall judgement: (1) the randomization process, (2) deviations from intended interventions, (3) missing outcome data, (4) measurement of the outcome, (5) selection of reported results, and (6) overall. The final assessment indicates either of “low risk,” “some concern,” or “high risk”^25^. For CCTs or CBAs, the risk of bias was assessed using the Risk of Bias In Non-randomized studies-of Interventions (ROBINS-I) tool. ROBINS-I included seven domains: (1) bias due to confounding factors, (2) bias in the classification of interventions, (3) bias in the selection of participants into the study (or into the analysis), (4) bias due to deviations from intended interventions, (5) bias due to missing data, (6) bias arising from the measurement of the outcome, and (7) bias in the selection of the reported result. Based on these domains, each study was judged as having “low risk of bias,” “moderate risk of bias,” “serious risk of bias,” or “critical risk of bias”^26^.

### Data analysis

Heterogeneity was tested using the Cochran Q and *I*^*2*^ tests. Heterogeneity was identified using the Cochran Q test *p* < 0.1 or *I*^*2*^ > 50%^27^. If the synthesized data were heterogeneous, a meta-analysis was performed using the random-effects Mantel–Haenszel model. Otherwise, a fixed-effects model was used^28^. For scales where lower scores indicated better sleep quality, the scores were reversed so that higher scores consistently reflected better sleep quality across the studies. The synthesized data were calculated using the standardized mean difference (SMD) and 95% confidence interval (95% CI). For studies that included three or more arms (e.g., control vs. aromatherapy vs. music), the sample size of the control group was divided by the number of intervention groups, whereas the mean and standard deviation of the control group remained unchanged^29^.

### Network meta-analysis

A Bayesian network meta-analysis was performed to determine the effectiveness of the intervention. Network meta-analysis allows for direct and indirect comparisons of more than two interventions. Additionally, the ranking probability can be calculated between the interventions to reveal the most effective interventions^30^. We performed the network meta-analysis according to the following procedure: (1) A network meta-analysis should meet the transitivity assumption among non-pharmacological interventions. Therefore, a network plot was constructed to illustrate the geometry and relationships between the interventions. (2) Whether the transitivity assumption was violated was evaluated by examining the consistency and similarity. Transitivity means that when A, B, and C are compared directly, if A is more effective than B and if B is more effective than C, then A is expected to be more effective than C. Consistency refers to the statistical agreement between direct and indirect comparisons, which were assessed using a global and local inconsistency test.

Similarities were qualitatively assessed for each selected study from a methodological perspective. Therefore, to ensure similarity, the extracted population, intervention, comparison, and outcome elements among the studies were assessed. (3) The pooled data were estimated as SMDs and 95% CIs for the interventions. (4) The ranking probability and surface under the cumulative ranking (SUCRA) were estimated to evaluate the relative position of the non-pharmacological interventions. Finally, (5) publication bias was assessed visually using funnel plots and statistically using Begg’s test. The significance level was set at *p* < 0.05. All statistical analyses were performed using Stata version 18.1 (StataCorp LLC, College Station, Texas, USA)^31^.

## Results

### Study selection

**Fig. 1:**
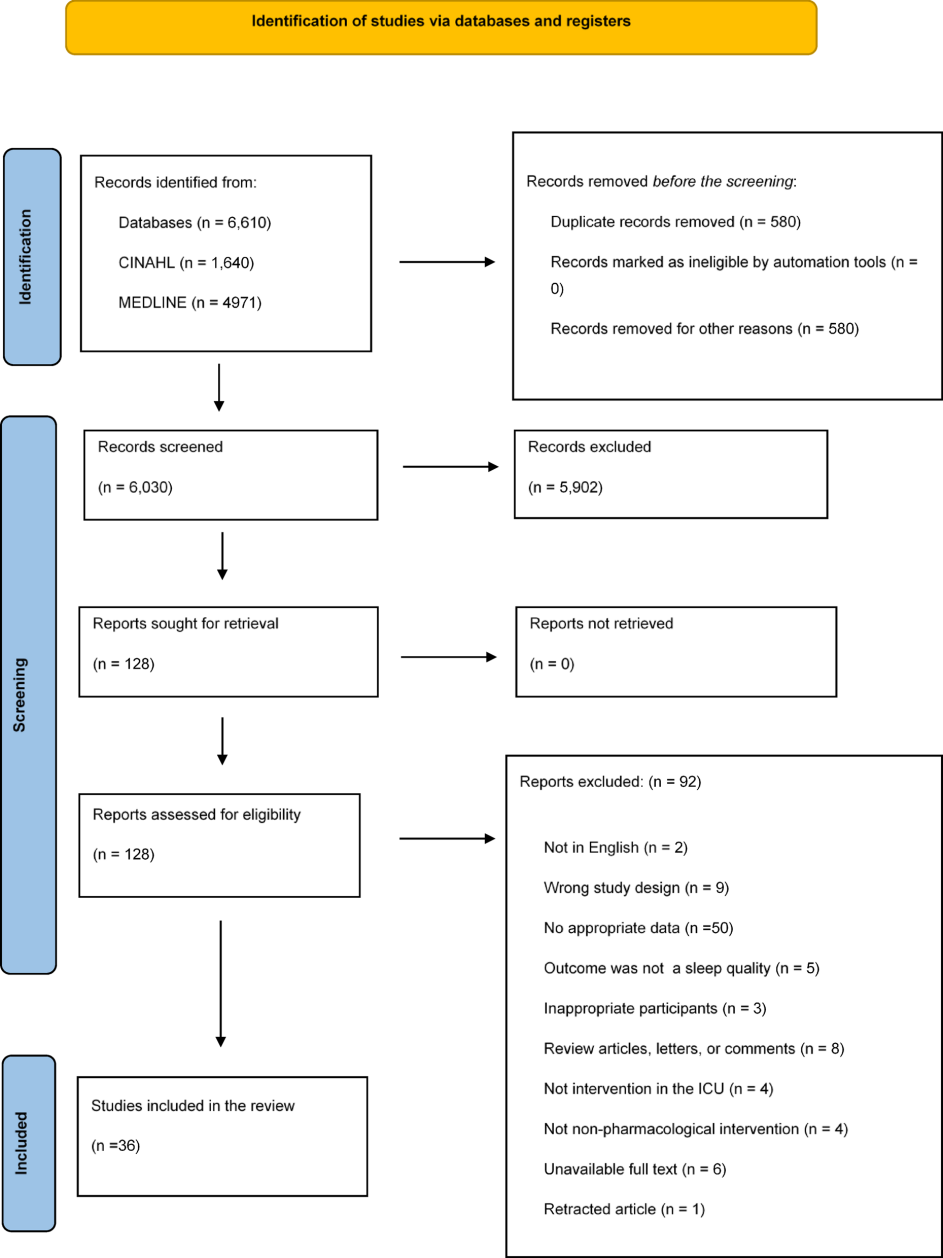
Flow diagram of the study identification, screening, review, and selection of studies based on the Preferred Reporting Items for Systematic Reviews and Meta-Analyses (PRISMA) 2020.

Figure 1 shows the research process for the articles, screening, and assessment of eligibility criteria based on the PRISMA study flow diagram^32^. The database search identified 6,610 records using the predefined search keywords. First, 580 duplicate studies were excluded. Second, we screened and removed 5,902 after analyzing the titles and abstracts in accordance with the inclusion and exclusion criteria. Finally, the remaining 128 studies were reviewed and their eligibility assessed. In conclusion, 36 articles met the eligibility criteria and were included in the network meta-analysis (Supplementary Table S3).

### Study characteristics and quality assessment

Table 2 shows the characteristics and content of non-pharmacological interventions performed to promote sleep. In terms of sleep assessments, 23 studies were assessed using the Richards–Campbell Sleep Questionnaire, four using the Pittsburgh Sleep Quality Index, five using the St. Mary’s Hospital Sleep Questionnaire, four using the Verran and Snyder–Halpern Sleep Scale, and one using the Stanford Sleepiness Scale. We extracted and categorized the intervention methods used in the included studies according to intervention type, including eye mask and earplugs (ten studies)^19,33–41^, aromatherapy (six studies)^8,42–46^, multicomponent (five studies)^47–51^, music (three studies)^52–54^, eye mask (three studies)^41,55,56^, valerian acupressure (two studies)^57,58^, earplugs (two studies)^41,59^, massage (two studies)^40,60^, ergonomic sleep mask, acupressure, preoperative orientation, family member presence, virtual reality, red light, eye mask and music, and comfort therapy (one study each)^34,57,61–66^. Details of the interventions performed in each study are presented in Supplemental Table S4.

**Table 2:** Characteristics of the studies (*n*=36).

Author, year	country	Population (Sample size)	Intervention	Comparison intervention	Age (mean ± SD, medium (IQR), or percentage)	study design	Measurement	Risk of bias
Aysun Kazak Saltı, 2024	Turkey	120 patients admitted to the ICU (CG, *n* = 30, IG: earplug, *n* = 30, eye mask, *n* = 30, both, *n* = 30)	1. Earplugs 2. Eye mask 3. Eye mask and earplugs	Usual care	CG: 52.83 ± 10.63 IG: earplug 57.66 ± 12.68 eye mask 58.66 ± 12.28 both 51.30 ± 16.19	RCT	RCSQ	High risk
Abolfazl Rahimi, 2022	Iran	100 cardiac surgery patients (48 CG, 52 IG)	Multicomponent interventions	Usual care	CG: 60.65 ± 10.035 IG: 60.6 ± 9.302	CCT	RCSQ	Critical
Ali Hajibagheri, 2014	Iran	60 cardiovascular patients (30 CG, 30 IG)	Aromatherapy	Usual care	CG: 63.9 ± 10.23 IG: 61.4 ± 11.64	RCT	PSQI	High risk
Ayyüce Tuba Koçak, 2021	Turkey	64 patitents admitted to medical neurology ICU (32 CG, 32 IG)	Eye mask and earplugs	Usual care	CG: 59.53 ± 17.94 IG: 59.37 ± 15.0	CBA	RCSQ	serious
Chiu-Ping Su, 2012	Taiwan	28 patients admitted to the ICU (14 CG, 14 IG)	Music therapy	Usual care	CG: 60.93 ± 10.78 IG: 62.43 ± 9.09	RCT	VSH Sleep Scale	Some concerns
Ebubekir Kaplan, 2025	Turkey	80 patitents admitted to medical CICU (40 CG, 40 IG)	Comfort therapy	Usual care	CG: 49.7 ± 9.17 IG: 49.07 ± 10.07	RCT	RCSQ	Some concerns
Emine Arık, 2020	Turkey	41 neurosurgery patients (20 CG, 21 IG)	Eye mask and earplugs	Usual care	CG: 48.5 (17–67) IG: 63 (31–72)	RCT	RCSQ	High risk
Eun Hee Cho, 2017	Korea	60 patients admitted to the ICU (30 CG, 30 IG)	Aromatherapy	Usual care	CG: 61.53 ± 8.8 IG: 59.5 ± 9.1	CBA	VSH Sleep Scale	Serious
Gülcan Bahcecioglu Turan, 2023	Turkey	70 heart failure patients (35 CG, 35 IG)	Eye mask and earplugs	Usual care	CG: 66.71 ± 8.92 IG: 65.4 ± 7.81	RCT	RSCQ	High risk
Hossein Bagheri, 2024	Iran	90 ACS patients (45 CG, 45 IG)	Family member presence	Usual care	CG: 63.58 ± 13.12 IG: 64.26 ± 13.95	RCT	SMHSQ	High risk
Jeongmin Kim, 2020	Korea	89 postoperative elderly patients (45 CG, 44 IG)	Music Therapy	Usual care	CG: 74.1 ± 6.7 IG: 74.6 ± 5.2	RCT	RCSQ	High risk
Ji-Han Chen, 2012	Taiwan	85 patients admitted to the ICU (44 CG, 41 IG)	Valerian acupressure	No valerian acupressure	CG: 69.1 ± 15.1 IG: 72.1 ± 18.2	RCT	SSS	High risk
Julián Díaz-Alonso, 2018	Spain	40 valve cardiac surgery patients (20 CG, 20 IG)	Preoperative orientation	Usual care	CG: <65 35%, 65–74 20%, ≥ 75 45% IG: <65 25%, 65–74 40%, ≥75 35%	RCT	RCSQ	Some concerns
Kurosh Jodaki, 2021	Iran	60 cardiovascular patients (30 CG, 30 IG)	Aromatherapy	Placebo	CG: 61.5 ± 12.75 IG: 62.8 ± 11.8	RCT	SMHSQ	Low risk
Lin Chen, 2022	China	67 patients underwent OPCABG surgery (37 CG, 30 IG)	Multicomponent interventions	Usual care	CG: 66.78 ± 8.08 IG: 64.97 ± 8.36	CCT	RCSQ	Serious
Mahin Moeini, 2010	Iran	64 cardiovascular patients (32 CG, 32 IG)	Aromatherapy	Usual care	CG: 52.8 ± 8.5 IG: 55.7 ± 7.7	RCT	SMHSQ	High risk
Masoumeh Bagheri-Nesami, 2015	Iran	60 acute coronary syndrome patients (30 CG, 30 IG)	Acupressure	Usual care	CG: 61.6 ± 10.52 IG: 60.3 ± 11.78	RCT	SMHSQ	Some concerns
Mi-Yeon Cho, 2013	Korea	56 patient underwent PCI (28 CG, 28 CG)	Aromatherapy	Usual care	CG: ≤60 53.6%, 61–70 32.1%, ≥ 71 14.3% IG: ≤60 28.6%, 61–70 42.8%, ≥ 71 28.6%	non-RCT	VSH Sleep Scale	Serious
Mohabat Habibi Nezhad, 2023	Iran	40 patients admitted to the ICU (20 CG, 20 CG)	Massage	Earplug and eye mask	CG: 45.5 ± 13.37 IG: 42.3 ± 12.65	RCT	RCSQ	High risk
Mohammad Daneshmandi, 2012	Iran	60 ACS patients (30 CG, 30 IG)	Eye mask	Usual care	N.A	RCT	PSQI	High risk
Mohammad K. Bani Younis, 2019	Jordan	103 patitents admitted to the ICU (51 CG, 52 IG)	Eye mask and earplugs	Usual care	CG: 56.18 ± 18.56 IG :51.18 ± 18.67	RCT	RCSQ	High risk
Nihal Topcu, 2022	Turkey	87 patients admitted to the ICU (49 CG, 38 IG)	Multicomponent interventions	Usual care	CG: 68.9 ± 12.8 IG: 73.3 ± 13.3	CBA	RCSQ	Serious
Nilofar Pasyar, 2024	Iran	80 AMI patients (40 CG, 40 IG)	Massage	Usual care	CG: 59.50 ± 8.02 IG: 57.42 ± 8.76	RCT	PSQI	High risk
Osamudiamen O Obanor, 2021	United states	87 breast free flap surgery patients (43 CG, 44 IG)	Eye mask and earplugs	Usual care	CG: 50.7 ± 8.7 IG: 51.4 ± 9.3	RCT	RCSQ	High risk
Öznur Kavaklı, 2023	Turkey	100 cardiovascular patients (50 CG, 50 IG)	Eye mask	Usual care	CG: 59.54 ± 10.73 IG: 56.78 ± 15.44	RCT	RCSQ	High risk
Pouya Farokhnezhad Afshar, 2016	Iran	60 patients admitted to the ICU (30 CG, 30 IG)	Music Therapy	Usual care	CG: 60.6 ± 11.53 IG: 58.87 ± 10.92	CBA	PSQI	Critical
Pureepat Arttawejkul, 2020	Thailand	17 patitents admitted to medical ICU (9 CG, 8 IG)	Eye mask and earplugs	Usual care	CG: 76 ± 32 IG: 67 ± 25	RCT	RCSQ	High risk
Reva Balci Akpinar, 2022	Turkey	84 patitents admitted to medical CICU (42 CG, 42 IG)	Eye mask and earplugs	Usual care	CG: 63.7 ± 6.92 IG: 64.04 ± 8.64	RCT	RCSQ	High risk
Seyed Mahdi Motahary, 2025	Iran	80 patients admitted to the CCU (40 CG, 40 IG)	Red light	Usual care	CG: 58.5 ± 11.83 IG: 59.7 ± 11.39	RCT	SMHSQ	High risk
Shu-Yen Li, 2011	Taiwan	55 postoperative patients (27 CG, 28 IG)	Multicomponent interventions	Usual care	CG: 50.7 ± 2.4 IG: 49.3 ± 6	CBA	RCSQ	Critical
Sibel Altintaş, 2023	Turkey	128 postoperative patients (64 CG, 64 IG)	Ergonomic sleep masks	Eye masks and earplugs	CG: 61.31 ± 14.87 IG: 66.42 ± 14.67	RCT	RSCQ	High risk
Soogyeong Kim, 2025	Korea	93 patitents admitted to the ICU (45 CG, 48 IG)	Virtual reality	Usual care	CG: 66.28 ± 12.46 IG: 67.63 ± 11.31	RCT	VSH	High risk
Tuğçe Topal, 2025	Turkey	45 abdominal surgery patients (24 CG, 21 IG)	Eye mask and music	Usual care	CG: 69.29 ± 13.5 IG: 63.23 ± 15.4	RCT	RCSQ	High risk
Yanting Zhang, 2024	China	246 patitents admitted to the ICU (121 CG, 125 IG)	Multicomponent interventions	Usual care	CG: 18–35 9.1%, 36–59 39.7%, ≥ 60 51.2% IG: 18–35 20.8%, 36–59 36.8%, ≥ 60 42.4%	CCT	RCSQ	Serious
Yun-Chian Lin, 2022	Taiwan	107 cardiovascular patients (52 CG, 55 IG)	Earplugs	Usual care	CG: 60.13 ± 13.21 IG: 57.92 ± 12.61	RCT	RCSQ	High risk
Zeynep Karaman Özlü, 2017	Turkey	60 postoperative patients (30 CG, 30 IG)	aromatherapy	Usual care	CG: 21–35 16.7%, 36–50 13.3%, > 51 70.0% IG: 21–35 23.3%, 36–50 23.3%, > 51 53.4%	CBA	RCSQ	Critical

ACS, acute coronary syndrome; AMI, acute myocardial infarction; CBA, controlled before and after study; CCT, controlled crinical trials; CCU, coronary care unit; CICU, coronary intensive care unit; CG, control group; ICU, intensive care unit; IG, intervention group; OPCABG, off-pump coronary artery bypass grafting; RCSQ, Richards–Campbell Sleep Questionnaire; RCT, randomized controlled trials; SMHSQ, St. Mary’s Hospital Sleep Questionnaire; SSS, Stanford Sleepiness Scale; VSH, Verran and Snyder–Halpern Sleep Scale.

For the quality assessment, 26 RCTs were evaluated using the RoB tool. In the overall domain in the RoB tool, 21 of the 26 studies (80.8%) were rated as “high risk,” one study (3.8%) as “low risk,” and four studies (15.4%) as “some concern.” However, non-RCTs, including CCTs and CBAs, were evaluated using ROBINS-I. Among them, six of the 10 studies (60.0%) were judged as “Serious” and four studies (40.0%) as “critical.” Quality assessment results evaluated using RoB and ROBINS-I for all included studies are detailed in Supplementary Table S5 and S6.

### Effectiveness of non-pharmacological interventions in sleep promotion

**Fig. 2:**
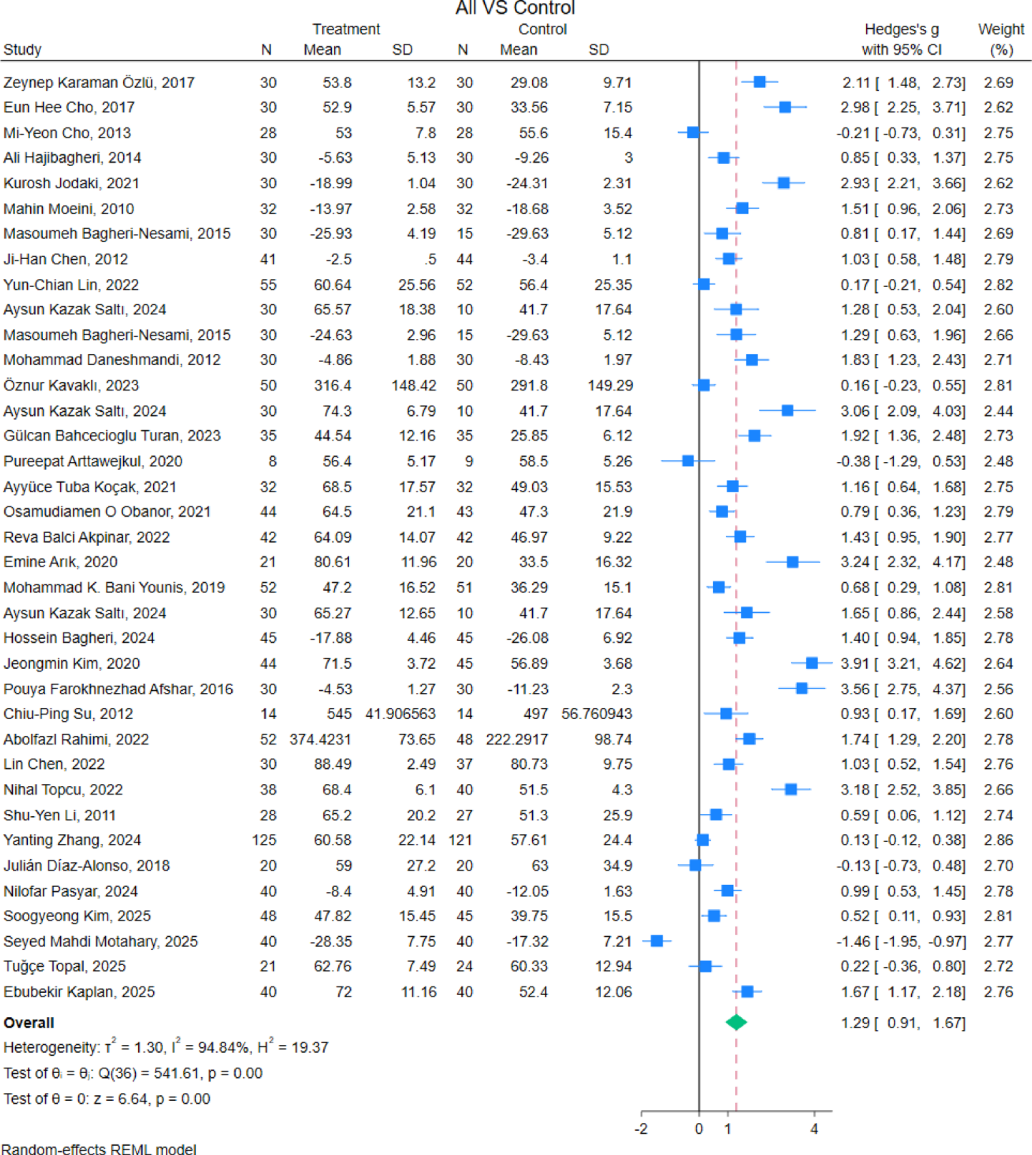
Forest plot showing the effects of non-pharmacological interventions on improving sleep. The forest plot indicates the sample size, effect size, and 95% confidence interval (95% CI) of each study, the weighted effect size and 95% CI. This includes the boxes, sample size; effect size; horizontal line, 95% CI; apex of the rhombus, weighted effect size; rhombus width, 95% CI. The pooled data from 36 trials of non-pharmacological interventions had a significant effect on improving sleep (standardized mean difference, 1.28; 95% CI, 0.90–1.65; *p* < 0.001), and heterogeneity was high because *I*^*2*^ was 94.67%, and the Cochran Q test showed *p* < 0.001.

The *I*^*2*^ test posited a heterogeneity of 94.7%, and the Cochran Q test showed *p* < 0.01, indicating high heterogeneity. Therefore, from the pooled data, the SMD and 95% CI were calculated using the random-effects Mantel–Haenszel model. In the pair-wise analysis, 34 studies were included, and two studies were excluded as they had no control group. Data from 2,619 participants were synthesized, among which 1,355 and 1,264 participants belonged to the intervention and control groups, respectively. As a result of the analysis using the random-effects Mantel–Haenszel model, non-pharmacological intervention significantly improved sleep quality (SMD, 1.29; 95% CI, 0.91 to 1.67; *p* < 0.01) (Fig. 2). We performed a visual inspection of the funnel plot and found that it was an indicated asymmetry; several studies deviated from the isosceles triangle in that there was publication bias (Supplementary Fig. S1). Furthermore, the rank correlation coefficient calculated using Begg’s test was significant (z = 3.44, *p* < 0.01).

**Fig. 3:**
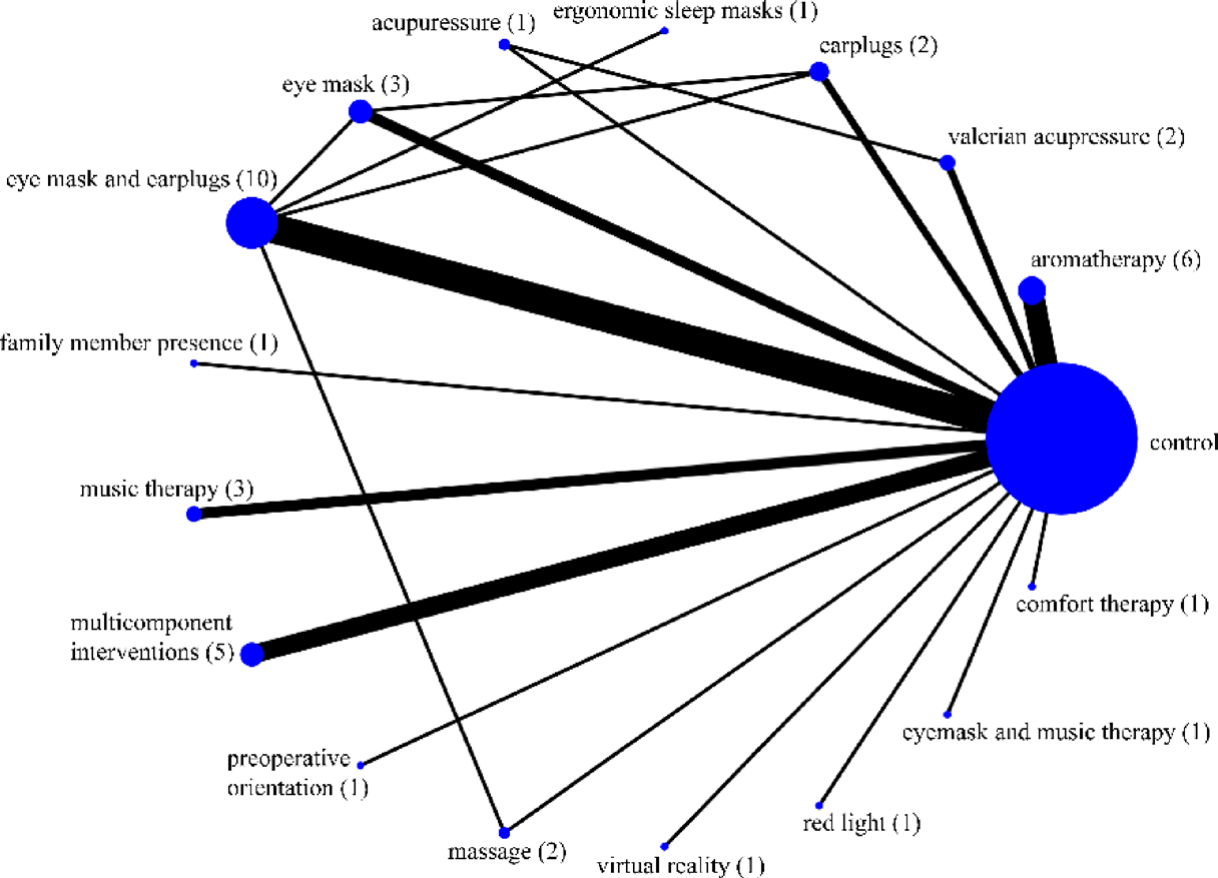
Network geometry of comparisons among non-pharmacological interventions and control conditions for improving sleep quality. The diagram illustrates six direct comparisons and five closed loops within the evidence network. The number of studies for each comparison is indicated by the thickness of the lines and by the number in brackets.

The network geometric was described as a network map. In addition to comparisons between the control and intervention groups, six direct comparisons were made among the intervention groups, and five closed loops were formed. The closed loops included valerian acupressure vs. acupressure, earplugs vs. eye mask and earplugs, earplugs vs. eye mask, ergonomic sleep masks vs. eye mask and earplugs, and eye mask and earplugs vs. massage. (Fig. 3). A total of 2,787 participants, 1,563 in the intervention group and 1224 in the control group, were pooled, and a network meta-analysis was performed.

**Fig. 4:**
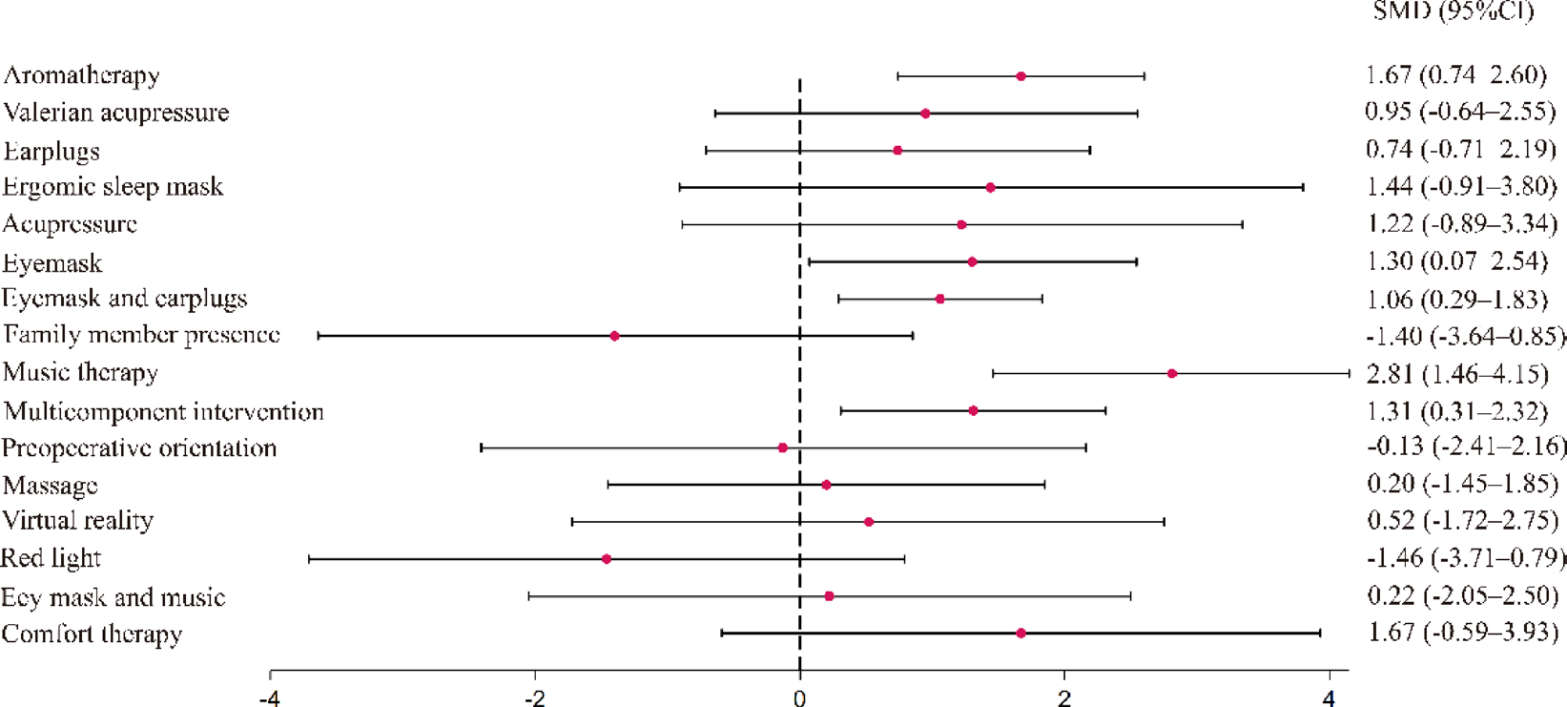
Forest plot of network meta-analysis to assess the effectiveness of improving sleep quality by non-pharmacological interventions.

There was no evidence of consistency in the results of conducting global (χ^2^ = 3.32, *p* = 0.65) and local inconsistency tests (all *p* > 0.05) to confirm inconsistency for closed loops (Supplementary Table S7). Figure 4 shows the results of the network meta-analysis comparing the intervention and control groups. Significant differences compared with the control groups were observed for aromatherapy (SMD = 1.67; 95% CI, 0.74 to 2.60; *p* < 0.01), eye mask (SMD = 1.30; 95% CI, 0.07 to 2.54; *p* = 0.04), eye mask and earplugs (SMD = 1.06; 95% CI, 0.29 to 1.83; *p* < 0.01), music therapy (SMD = 2.81; 95% CI, 1.46 to 4.15; *p* < 0.01), and multicomponent intervention (SMD = 1.31; 95% CI, 0.31 to 2.32; *p* = 0.01 ). According to SUCRA analysis, music therapy (94.9%) showed the highest probability of being the most effective non-pharmacological intervention for improving sleep, followed by aromatherapy (76.2%). The mean ranks were 1.8 for music therapy and 4.8 for aromatherapy (Table 3).

**Table 3:** Ranking probabilities and surface under the cumulative ranking curve (SUCRA) for non-pharmacological interventions to improve sleep. Ranking probabilities indicate the likelihood of each intervention achieving each possible rank. SUCRA presents the rank of effect Estimation for each intervention. SUCRA ranges from 0% to 100%, with higher values indicating a greater probability of being the best treatment. A value of 100% indicates that the treatment is certain to be the best, whereas 0% indicates that it is certain to be the worst.

Control	Mean rank	SUCRA (%)
	12.9	25.7
Aromatherapy	4.8	76.2
Valerian acupressure	8.3	54.4
Earplugs	9.2	48.6
Ergonomic sleep masks	6.4	66.0
Acupressure	7.2	61.2
Eye mask	6.5	65.4
Eye mask and earplugs	7.7	58.0
Family member presence	15.5	9.6
Music therapy	1.8	94.9
Multicomponent intervention	6.5	65.6
Preoperative orientation	12.3	29.5
Massage	11.5	34.4
Virtual reality	10	43.7
Red light	15.6	9.0
Eye mask and music	11.1	36.9
Comfort therapy	5.6	70.9

## Discussion

This study revealed effective non-pharmacological intervention for improving sleep in patients in the ICU through a systematic review and network meta-analysis. Thirty-six studies, including both randomized and non-RCTs, comprising 2619 patients, were included. The included studies were geographically concentrated in Asian and Middle Eastern countries. In a network meta-analysis, five types of interventions, including aromatherapy, eye mask, eye mask and earplugs, music therapy, and multicomponent interventions, were effective in improving sleep in patients in the ICU. Music therapy was the most effective non-pharmacological intervention for improving sleep, followed by aromatherapy. Consequently, it is assumed that interrupting sensory stimulation, such as light and noise, or stabilizing autonomic nervous system and neurotransmitter is crucial to improving sleep in patients in the ICU. Furthermore, multicomponent interventions that combine these interventions may have had synergistic effects and relate to improved sleep.

Sleep–wake regulation is related to several neurological pathways, such as adrenergic, GABAergic, and cholinergic neurons, orexin, and histamine, which are located in the hypothalamic area and brainstem reticular formation^67^. GABAergic neurons located in the ventral lateral preoptic area (VLPO) of the hypothalamus are inhibitors of neurotransmitters in the brainstem and hypothalamus that are activated by somnogenic factors, such as adenosine. These neurons promote and maintain sleep by suppressing excitatory monoaminergic and orexinergic neurons in the hypothalamus, which was an excitatory neuron^68–70^.

Orexin neurons project to brain regions including the locus coeruleus (LC), dorsal raphe nucleus, and tuberomammillary area of the brainstem, activating noradrenergic, serotoninergic, and histaminergic neurons, and play a crucial role in maintaining arousal by indirectly inhibiting GABAergic neurons in the VLPO^67,71^. As orexin neurons widely receive inputs from the limbic system, the suprachiasmatic nucleus (SCN) interacts with systems that regulate emotion and circadian rhythms to maintain proper vigilance states^72,73^. Noradrenergic neurons are located in the LC project LHA, VLPO, and cerebral cortex^74^, which promote and maintain the awake state by activating orexinergic neurons, inhibiting GABAergic neurons, and releasing them into the cerebral cortex. In addition, LC neurons are activated by sensory stimulation, such as visual, auditory, and somatosensory stimulation, and increase noradrenaline release^75,76^.

In addition to these neurotransmitter systems, melatonin plays a central role in the regulation of circadian rhythms, core body temperature, and the sleep–wake cycle. Melatonin is secreted by the pineal gland under the control of the SCN. Melatonin secretion promotes sleep by dilating the peripheral vasculature, decreasing core body temperature^77^, and suppressing sympathetic vasomotor tone by enhancing GABA_A_ receptor activity^78^. However, melatonin secretion is suppressed by light exposure at night, particularly at blue light wavelengths^79^.

Non-pharmacological interventions for sleep improvement, such as music and aromatherapy, may modulate this physiological mechanism. Aromatherapy is commonly administered through the inhalation of scent, and sensory signals from the olfactory nervous system project to the hypothalamus, basal ganglia, and hippocampus via the olfactory bulb and upper olfactory cortex. Consequently, the scent of aromatherapy sends signals to the brain, affecting neurotransmitter regulation and autonomic nervous system activity related to sleep^80^. In this study, four trials used lavender oil, and two used Rosa damascene. Linalool acetate, which includes these aromas, activates the parasympathetic nerve^81,82^. In animal studies, the inhalation of lavender has been reported to evoke GABA and increase delta waves on electroencephalography (EEG), as indicated by deep sleep^83,84^. Therefore, aromatherapy may improve sleep in patients in the ICU by modulating neurophysiological mechanisms. Classical and rhythmic music with a tempo of 60–80 beats/min are frequently used as music therapy. Two main central nervous system components are involved in the stress response. Corticotropin-releasing hormone, secreted by the hypothalamus, stimulates adrenocorticotropic hormone release and elevates plasma cortisol levels^85^. In contrast, the autonomic nervous system stimulates norepinephrine release from the LC and activates the sympathetic nervous system^86^. Acoustic stimuli through music therapy decrease cortisol and catecholamine levels and activate the parasympathetic nervous system via the hypothalamic–brainstem autonomic axis^87–89^.

In addition, eye masks and earplugs can effectively block light and noise stimulation. Because patients in the ICU receive interventions, such as nursing care, to detect early abnormalities and sustain their lives, the noise and light levels in the ICU are sufficiently high to disrupt sleep. The mean noise level in the ICU is approximately 60 dB, which exceeds the nighttime noise level recommended by the World Health organization^90,91^. Noise stimulation activates noradrenergic neurons in the LC, whereas light stimulation decreases melatonin secretion and induces sleep disruption. Therefore, the use of earplugs and eye masks may improve sleep in patients in the ICU by interrupting these stimuli.

Patients in the ICU experience high stress due to invasive treatments and various ICU environments. Additionally, the occurrence of delirium is high and is associated with sleep^92^. Therefore, ensuring adequate sleep is essential for preventing delirium. Many effective non-pharmacological interventions identified in this review, such as eye masks, earplugs, aromatherapy, and music therapy, are relatively easy to implement and require limited resources. Furthermore, from a physiological standpoint, sensory interruption by light and noise or stabilization of the autonomic nerve and neurotransmitters may be effective in improving sleep. Because these non-pharmacological interventions are implemented from bedtime to waking, nurses—who provide continuous bedside care 24/7—are ideally positioned to implement them. Therefore, nurses are expected to play central and active roles in implementing these strategies in clinical settings.

This review has some limitations. First, although a systematic search was conducted, the search strategy was developed by the authors without formal input from an information specialist, and controlled vocabulary (e.g., MeSH terms) was not systematically applied. As a result, some relevant studies may have been missed. Second, a geographical bias was present, as the included studies were concentrated in Asian and Middle Eastern countries. Therefore, the generalizability of the findings to ICUs with different baseline care practices should be interpreted with caution. Third, the review included various types of ICUs and participant characteristics, resulting in substantial heterogeneity across the analyses. Finally, most of the included studies had a low methodological quality because blinding was not feasible when sleep outcomes were assessed using self-reported questionnaires. Consequently, most studies were judged to be at high risk of bias. Therefore, future studies should incorporate objective sleep assessments, including EEG, to increase the reliability of these findings.

In conclusion, this systematic review and network meta-analysis revealed effective non-pharmacological interventions for improving sleep in patients in the ICU. Aromatherapy, music therapy, eye masks, earplugs, and multicomponent interventions effectively improve sleep. From a physiological perspective, these non-pharmacological interventions may improve sleep by reducing exposure to environmental light and noise and the stabilization of autonomic nervous system activity and neurotransmitter regulation. Consequently, based on these findings, nurses should play a central role in improving sleep quality in patients in the ICU.

## Supplementary Information

Below is the link to the electronic supplementary material.

Supplementary Material 1

Supplementary Material 2

## Data Availability

The datasets collected in this study are available from the corresponding author upon reasonable request.
