# Supplementary material for: Effects of non-pharmacological interventions on sleep in patients with critical illness: a systematic review and network meta-analysis
Source: Sci Rep. 2026 Feb 9;16:7883. doi: 10.1038/s41598-026-39187-y (PMC12954107; doi:10.1038/s41598-026-39187-y)
Supplement: Supplementary file 1 — Supplementary Material 1 [file 41598_2026_39187_MOESM1_ESM.docx]

**Supplementary Table S1 Preferred Reporting Items for Systematic Reviews and Meta-Analyses (PRISMA) Network Meta-Analyses checklist.**

| **Section/Topic** | **Item #** | **Checklist Item** | **Reported on Page #** |
| --- | --- | --- | --- |
| **TITLE** |  |  |  |
| Title | 1 | Identify the report as a systematic review *incorporating a network meta-analysis (or related form of meta-analysis).* | ***Page 1*** |
|  |  |  |  |
| **ABSTRACT** |  |  |  |
| Structured summary | 2 | Provide a structured summary including, as applicable:  **Background:** main objectives  **Methods:** data sources; study eligibility criteria, participants, and interventions; study appraisal; and *synthesis methods, such as network meta-analysis.*  **Results:** number of studies and participants identified; summary estimates with corresponding confidence/credible intervals; *treatment rankings may also be discussed. Authors may choose to summarize pairwise comparisons against a chosen treatment included in their analyses for brevity.*  **Discussion/Conclusions:** limitations; conclusions and implications of findings.  **Other:** primary source of funding; systematic review registration number with registry name. | ***Page 2*** |
|  |  |  |  |
| **INTRODUCTION** |  |  |  |
| Rationale | 3 | Describe the rationale for the review in the context of what is already known*, including mention of why a network meta-analysis has been conducted.* | ***Page 3-4*** |
| Objectives | 4 | Provide an explicit statement of questions being addressed, with reference to participants, interventions, comparisons, outcomes, and study design (PICOS). | ***Page 4*** |
|  |  |  |  |
| **METHODS** |  |  |  |
| Protocol and registration | 5 | Indicate whether a review protocol exists and if and where it can be accessed (e.g., Web address); and, if available, provide registration information, including registration number. | ***Page 5*** |
| Eligibility criteria | 6 | Specify study characteristics (e.g., PICOS, length of follow-up) and report characteristics (e.g., years considered, language, publication status) used as criteria for eligibility, giving rationale. *Clearly describe eligible treatments included in the treatment network, and note whether any have been clustered or merged into the same node (with justification).* | ***Page 5-6*** |
| Information sources | 7 | Describe all information sources (e.g., databases with dates of coverage, contact with study authors to identify additional studies) in the search and date last searched. | ***Page 5*** |
| Search | 8 | Present full electronic search strategy for at least one database, including any limits used, such that it could be repeated. | ***Page 5***  ***Table 1*** |
| Study selection | 9 | State the process for selecting studies (i.e., screening, eligibility, included in systematic review, and, if applicable, included in the meta-analysis). | ***Page 6*** |
| Data collection process | 10 | Describe method of data extraction from reports (e.g., piloted forms, independently, in duplicate) and any processes for obtaining and confirming data from investigators. | ***Page 6*** |
| Data items | 11 | List and define all variables for which data were sought (e.g., PICOS, funding sources) and any assumptions and simplifications made. | ***Page 6*** |
| **Geometry of the network** | **S1** | Describe methods used to explore the geometry of the treatment network under study and potential biases related to it. This should include how the evidence base has been graphically summarized for presentation, and what characteristics were compiled and used to describe the evidence base to readers. | ***Page 7-8*** |
| Risk of bias within individual studies | 12 | Describe methods used for assessing risk of bias of individual studies (including specification of whether this was done at the study or outcome level), and how this information is to be used in any data synthesis. | ***Page 6*** |
| Summary measures | 13 | State the principal summary measures (e.g., risk ratio, difference in means). *Also describe the use of additional summary measures assessed, such as treatment rankings and surface under the cumulative ranking curve (SUCRA) values, as well as modified approaches used to present summary findings from meta-analyses.* | ***Page 7*** |
| Planned methods of analysis | 14 | Describe the methods of handling data and combining results of studies for each network meta-analysis. This should include, but not be limited to:   - *Handling of multi-arm trials;* - *Selection of variance structure;* - *Selection of prior distributions in Bayesian analyses; and* - *Assessment of model fit.* | ***Page 7-8*** |
| **Assessment of Inconsistency** | **S2** | Describe the statistical methods used to evaluate the agreement of direct and indirect evidence in the treatment network(s) studied. Describe efforts taken to address its presence when found. | ***Page 7-8*** |
| Risk of bias across studies | 15 | Specify any assessment of risk of bias that may affect the cumulative evidence (e.g., publication bias, selective reporting within studies). | ***Page 8*** |
| Additional analyses | 16 | Describe methods of additional analyses if done, indicating which were pre-specified. This may include, but not be limited to, the following:   - Sensitivity or subgroup analyses; - Meta-regression analyses; - *Alternative formulations of the treatment network; and* - *Use of alternative prior distributions for Bayesian analyses (if applicable).* | ***N.A.*** |
|  |  |  |  |
| **RESULTS†** |  |  |  |
| Study selection | 17 | Give numbers of studies screened, assessed for eligibility, and included in the review, with reasons for exclusions at each stage, ideally with a flow diagram. | ***Page 8***  ***Figure 1*** |
| **Presentation of network structure** | **S3** | Provide a network graph of the included studies to enable visualization of the geometry of the treatment network. | ***Page 10***  ***Figure 3*** |
| **Summary of network geometry** | **S4** | Provide a brief overview of characteristics of the treatment network. This may include commentary on the abundance of trials and randomized patients for the different interventions and pairwise comparisons in the network, gaps of evidence in the treatment network, and potential biases reflected by the network structure. | ***Page 10*** |
| Study characteristics | 18 | For each study, present characteristics for which data were extracted (e.g., study size, PICOS, follow-up period) and provide the citations. | ***Page 8-9***  ***Table 2*** |
| Risk of bias within studies | 19 | Present data on risk of bias of each study and, if available, any outcome level assessment. | ***Page 9***  ***S2 and S3*** |
| Results of individual studies | 20 | For all outcomes considered (benefits or harms), present, for each study: 1) simple summary data for each intervention group, and 2) effect estimates and confidence intervals. *Modified approaches may be needed to deal with information from larger networks.* | ***Page 8-9*** |
| Synthesis of results | 21 | Present results of each meta-analysis done, including confidence/credible intervals. *In larger networks, authors may focus on comparisons versus a particular comparator (e.g. placebo or standard care), with full findings presented in an appendix. League tables and forest plots may be considered to summarize pairwise comparisons.* If additional summary measures were explored (such as treatment rankings), these should also be presented. | ***Page 9-10***  ***Figure 2*** |
| **Exploration for inconsistency** | **S5** | Describe results from investigations of inconsistency. This may include such information as measures of model fit to compare consistency and inconsistency models, *P* values from statistical tests, or summary of inconsistency estimates from different parts of the treatment network. | ***Page 10***  ***S5*** |
| Risk of bias across studies | 22 | Present results of any assessment of risk of bias across studies for the evidence base being studied. | ***Page 9-10*** |
| Results of additional analyses | 23 | Give results of additional analyses, if done (e.g., sensitivity or subgroup analyses, meta-regression analyses*, alternative network geometries studied, alternative choice of prior distributions for Bayesian analyses,* and so forth). | ***N.A.*** |
|  |  |  |  |
| **DISCUSSION** |  |  |  |
| Summary of evidence | 24 | Summarize the main findings, including the strength of evidence for each main outcome; consider their relevance to key groups (e.g., healthcare providers, users, and policy-makers). | ***Page 11*** |
| Limitations | 25 | Discuss limitations at study and outcome level (e.g., risk of bias), and at review level (e.g., incomplete retrieval of identified research, reporting bias). *Comment on the validity of the assumptions, such as transitivity and consistency. Comment on any concerns regarding network geometry (e.g., avoidance of certain comparisons).* | ***Page 14*** |
| Conclusions | 26 | Provide a general interpretation of the results in the context of other evidence, and implications for future research. | ***Page 14-15*** |
|  |  |  |  |
| **FUNDING** |  |  |  |
| Funding | 27 | Describe sources of funding for the systematic review and other support (e.g., supply of data); role of funders for the systematic review. This should also include information regarding whether funding has been received from manufacturers of treatments in the network and/or whether some of the authors are content experts with professional conflicts of interest that could affect use of treatments in the network. |  |

PICOS = population, intervention, comparators, outcomes, study design.

* Text in italics indicateS wording specific to reporting of network meta-analyses that has been added to guidance from the PRISMA statement.

† Authors may wish to plan for use of appendices to present all relevant information in full detail for items in this section.

**Supplementary Table S2 PRISMA-S Checklist**

| **Section/topic** | **#** | **Checklist item** | **Location(s) Reported** |
| --- | --- | --- | --- |
| **INFORMATION SOURCES AND METHODS** | | | |
| Database name | 1 | Name each individual database searched, stating the platform for each. | Page 5 |
| Multi-database searching | 2 | If databases were searched simultaneously on a single platform, state the name of the platform, listing all of the databases searched. | Page 5 |
| Study registries | 3 | List any study registries searched. | Page 5 |
| Online resources and browsing | 4 | Describe any online or print source purposefully searched or browsed (e.g., tables of contents, print conference proceedings, web sites), and how this was done. | Not |
| Citation searching | 5 | Indicate whether cited references or citing references were examined, and describe any methods used for locating cited/citing references (e.g., browsing reference lists, using a citation index, setting up email alerts for references citing included studies). | Not |
| Contacts | 6 | Indicate whether additional studies or data were sought by contacting authors, experts, manufacturers, or others. | Not |
| Other methods | 7 | Describe any additional information sources or search methods used. | Not |
| **SEARCH STRATEGIES** | | | |
| Full search strategies | 8 | Include the search strategies for each database and information source, copied and pasted exactly as run. | Table 1 |
| Limits and restrictions | 9 | Specify that no limits were used, or describe any limits or restrictions applied to a search (e.g., date or time period, language, study design) and provide justification for their use. | Page 6 |
| Search filters | 10 | Indicate whether published search filters were used (as originally designed or modified), and if so, cite the filter(s) used. | Page 6 |
| Prior work | 11 | Indicate when search strategies from other literature reviews were adapted or reused for a substantive part or all of the search, citing the previous review(s). | Not |
| Updates | 12 | Report the methods used to update the search(es) (e.g., rerunning searches, email alerts). | Not |
| Dates of searches | 13 | For each search strategy, provide the date when the last search occurred. | Page 6 |
| **PEER REVIEW** | | | |
| Peer review | 14 | Describe any search peer review process. | Page 6, 7 |
| **MANAGING RECORDS** | | | |
| Total Records | 15 | Document the total number of records identified from each database and other information sources. | Page 9 |
| Deduplication | 16 | Describe the processes and any software used to deduplicate records from multiple database searches and other information sources. | Page 9 |
|  |  |  |  |
| PRISMA-S: An Extension to the PRISMA Statement for Reporting Literature Searches in Systematic Reviews | | |  |
| Rethlefsen ML, Kirtley S, Waffenschmidt S, Ayala AP, Moher D, Page MJ, Koffel JB, PRISMA-S Group. | | |  |
| Last updated February 27, 2020. | |  |  |

**Supplementary Table S4 Detailed description of non-pharmacological sleep-promoting interventions.**

| ***Author, year*** | ***Intervention*** | ***Components*** | ***Timing*** | ***Duration*** | ***Frequency*** |
| --- | --- | --- | --- | --- | --- |
| ***Aysun Kazak Saltı, 2024*** | 1. Earplugs 2. Eye mask 3. Eye mask and earplugs | 1. Earplugs 2. Eye mask 3. Eye mask and earplugs | At bedtime on night 2 | Overnight | Once |
| ***Abolfazl Rahimi, 2022*** | Multicomponent interventions | Noise reduction  Nursing care modifications  Using blanket Lighting turn off Eye mask and earplugs | At bedtime on night 1 and 2 | overnight (22:00–06:00) | For 2 consecutive nights |
| ***Ali Hajibagheri, 2014*** | Aromatherapy | Rosa damascena aromatherapy (3 drops on paper towel placed beside pillow) | At bedtime on night 2 | Overnight (21:00 - 06:00) | Once |
| ***Ayyüce Tuba Koçak, 2021*** | Eye mask and earplugs | Eye mask and earplugs | At bedtime on night 2 | Overnight | Once |
| ***Chiu-Ping Su, 2012*** | Music Therapy | Sedating music | At bedtime on night 3 (21:30–23:30) | 45 mins | Once |
| ***Ebubekir Kaplan, 2025*** | Comfort therapy | Care based on Kolcaba's comfort theory | Not reported | Not reported | During ICU stay |
| ***Emine Arık, 2020*** | Eye mask and earplugs | Eye mask and earplugs | At bedtime | Overnight (23:00–05:00) | Once nightly |
| ***Eun Hee Cho, 2017*** | Aromatherapy | Lavender essential oil (3 drops on aromastone hung on the centre of the bedside) | At bedtime (21:00) | Overnight (21:00–08:00) | For 2 consecutive nights |
| ***Gülcan Bahcecioglu Turan, 2023*** | Eye mask and earplugs | Eye mask and earplugs | At bedtime | Overnight (22:30–06:30) | For 3 consecutive nights |
| ***Hossein Bagheri, 2024*** | Family member presence | Increasing duration of family member's presence | From second to fourth day (15:00–21:00) | Two hours | Twice the day |
| ***Jeongmin Kim, 2020*** | Music Therapy | Patient-directed interactive music Passive music | Patient-directed interactive music: Daytime on day 2 (10:00–12:00) Passive music: At night on night 2 | Patient-directed interactive music: 15mins Passive music: 30 mins | Once |
| ***Ji-Han Chen, 2012*** | Valerian acupressure | Valerian acupressure | On night 2 (19:00–21:00) | 18 mins | Once |
| ***Julián Díaz-Alonso, 2018*** | Preoperative orientation | Explained the professionals’ assistance  including the common procedures and communication difficulties talking about the presumable sense of fear or uncertainty regarding clinical evolution. | Prior to ICU admission | 45–60 mins | Once |
| ***Kurosh Jodaki, 2021*** | Aromatherapy | Rosa damascena aromatherapy (5 drops on napkin attached shirt collar) | At bedtime on night 2 | Overnight (22:00–06:00) | Once |
| ***Lin Chen, 2022*** | Multicomponent interventions | Light exposure and reduction Adapt ventilation mode Smooth and light music Back massage Eye mask and earplugs Noise reduction Avoid nocturnal interventions | Light exposure: daytime Light reduction: at bedtime Smooth and light music: at bedtime (21:00) Back massage: at bedtime Eye mask and earplugs: at bedtime (21:00) Noise reduction: at bedtime Avoid nocturnal interventions: at bedtime | Light exposure: 2–5 hours Light reduction: overngiht Smooth and light music: 30–45 mins Back massage: 6 mins Eye mask and earplugs: overnight Noise reduction: overnight Avoid nocturnal interventions: overnight | For 2 consecutive nights |
| ***Mahin Moeini, 2010*** | Aromatherapy | Lavender oil (cotton placed in a small box near patient's pillow) | At bedtime (21:00) | Overnight (21:00–06:00) | For 3 consecutive nights |
| ***Masoumeh Bagheri-Nesami, 2015*** | Acupressure | Acupressure | On night (19:00–21:00) | 18 mins | For 3 consecutive days |
| ***Mi-Yeon Cho, 2013*** | Aromatherapy | lavender, roman chamomile and neroli oil (aromastone placed under the patient's pillow) | At bedtime after PCI | Overnight | Once |
| ***Mohabat Habibi Nezhad, 2023*** | Massage | Swedish massage | At bedtime on night 2 (22:00) | 20 mins | Once |
| ***Mohammad Daneshmandi, 2012*** | Eye mask | Eye mask | At bedtime on night 2 | Overnight | Once |
| ***Mohammad K. Bani Younis, 2019*** | Eye mask and earplugs | Eye mask and earplugs | At bedtime on night 2 | Overnight (22:00–06:00) | Once |
| ***Nihal Topcu, 2022*** | Multicomponent interventions | Light Reduction Sound Reduction Disturbance Reduction Eye mask and earplugs | At bedtime on night 1 | Overnight (23:00–07:00) | Once |
| ***Nilofar Pasyar, 2024*** | Massage | Foot massage | Afternoon (16:00) | 15 mins | For 3 consecutive days |
| ***Osamudiamen O Obanor, 2021*** | Eye mask and earplugs | Eye mask and earplugs | At bedtime on night 1 | Overnight | Once |
| ***Öznur Kavaklı, 2023*** | Eye mask | Eye mask | At bedtime on night 2(22:30) | Overnight (22:30–06:30) | Once |
| ***Pouya Farokhnezhad Afshar, 2016*** | Music Therapy | White noise | At bedtime (day 4, 20:00–21:00 and 23:00–24:00) | One hour | For 3 consecutive nights |
| ***Pureepat Arttawejkul, 2020*** | Eye mask and earplugs | Eye mask and earplugs | At bedtime (habitual home bedtime) | Overnight (until 07:00) | For 5 consecutive nights |
| ***Reva Balci Akpinar, 2022*** | Eye mask and earplugs | Eye mask and earplugs | At bedtime on night 2 and 3 (22:30) | Overnight (22:30–06:30) | For 2 consecutive nights |
| ***Seyed Mahdi Motahary, 2025*** | Red light | Red light | At bedtime on night 2 and 3 (22:00) | Overnight (22:00–06:00) | For 2 consecutive nights |
| ***Shu-Yen Li, 2011*** | Multicomponent interventions | Noise reduction Light reduction Avoid nocturnal intervention | At bedtime | Overnight (23:00–05:00) | Once |
| ***Sibel Altintaş, 2023*** | Ergonomic sleep masks | a material that covers the eyes and earscompletely, | At bedtime on night 3 (22:00) | Overnight (22:00–06:00) | Once |
| ***Soogyeong Kim, 2025*** | Virtual reality | Mindfulness and relaxation mediation content | At bedtime (around 21:00) | 20 mins | Once |
| ***Tuğçe Topal, 2025*** | Eye mask and music | Eye mask and classical music | At bedtime (22:00) | Eye mask: Overnight Music: one hour | For 3 consecutive nights |
| ***Yanting Zhang, 2024*** | Multicomponent interventions | Noise reduction Eye mask and earplugs Light reduction Avoid nocturnal intervention | At bedtime on first night (22:00) | Overnight (22:00–07:00) | Once |
| ***Yun-Chian Lin, 2022*** | Earplugs | Earplugs | At bedtime on night 2 (22:00) | Overnight (22:00–07:00) | Once |
| ***Zeynep Karaman Özlü, 2017*** | Aromatherapy | Lavender oil | At bedtime (22:00) | 10–15 mins | Once |

**Supplementary Table S5 Assessment of risk of bias in randomized controlled trials using the Cochrane Risk of Bias tool.**

| **Study ID** | **D1** | **D2** | **D3** | **D4** | **D5** | **Overall** |
| --- | --- | --- | --- | --- | --- | --- |
| **Mohabat Habibi Nezhad, 2023** |  |  |  |  |  |  |
| **Hossein Bagheri, 2024** |  |  |  |  |  |  |
| **Mohammad Daneshmandi, 2012** |  |  |  |  |  |  |
| **Yun-Chian Lin, 2022** |  |  |  |  |  |  |
| **Reva Balci Akpinar, 2022** |  |  |  |  |  |  |
| **Ebubekir Kaplan, 2025** |  |  |  |  |  |  |
| **Tuğçe Topal, 2025** |  |  |  |  |  |  |
| **Nilofar Pasyar, 2024** |  |  |  |  |  |  |
| **Aysun Kazak Saltı, 2024** |  |  |  |  |  |  |
| **Soogyeong Kim, 2025** |  |  |  |  |  |  |
| **Seyed Mahdi Motahary, 2025** |  |  |  |  |  |  |
| **Öznur Kavaklı, 2023** |  |  |  |  |  |  |
| **Julián Díaz-Alonso, 2018** |  |  |  |  |  |  |
| **Ji-Han Chen, 2012** |  |  |  |  |  |  |
| **Ali Hajibagheri, 2014** |  |  |  |  |  |  |
| **Kurosh Jodaki, 2021** |  |  |  |  |  |  |
| **Emine Arık, 2020** |  |  |  |  |  |  |
| **Chiu-Ping Su, 2012** |  |  |  |  |  |  |
| **Mahin Moeini, 2010** |  |  |  |  |  |  |
| **Sibel Altintaş, 2023** |  |  |  |  |  |  |
| **Gülcan Bahcecioglu Turan, 2023** |  |  |  |  |  |  |
| **Mohammad K. Bani Younis, 2019** |  |  |  |  |  |  |
| **Pureepat Arttawejkul, 2020** |  |  |  |  |  |  |
| **Jeongmin Kim, 2020** |  |  |  |  |  |  |
| **Masoumeh Bagheri-Nesami, 2015** |  |  |  |  |  |  |
| **Osamudiamen O Obanor, 2021** |  |  |  |  |  |  |

|  |  |
| --- | --- |
| \|  \| \| --- \| | Low risk |
| \|  \| \| --- \| | Some concerns |
| \|  \| \| --- \| | High risk |

| D1 | Randomization process |  |
| --- | --- | --- |
| D2 | Deviations from the intended interventions |  |
| D3 | Missing outcome data |  |
| D4 | Measurement of the outcome |  |
| D5 | Selection of the reported result |  |

**Supplementary Table S6 Assessment of risk of bias in nonrandomized controlled trials using the Risk of Bias in Nonrandomized Studies of Interventions tool.**

|  | ***Confounding*** | ***Classification of interventions*** | ***Slection of pariticipants*** | ***Deviations from interventions*** | ***Missing data*** | ***Measurement of outcome*** | ***Selection of reported result*** | ***overall*** |
| --- | --- | --- | --- | --- | --- | --- | --- | --- |
| ***Eun Hee Cho, 2017*** | low | low | low | low | low | moderate | serious | serious |
| ***Mi-Yeon Cho, 2013*** | low | serious | serious | low | low | moderate | low | serious |
| ***Pouya Farokhnezhad Afshar, 2016*** | critical | low | low | low | low | moderate | low | critical |
| ***Yanting Zhang , 2024*** | serious | low | low | moderate | low | moderate | low | serious |
| ***Lin Chen, 2022*** | low | low | low | low | low | moderate | serious | serious |
| ***Nihal Topcu, 2022*** | serious | low | low | low | low | moderate | low | serious |
| ***Zeynep Karaman Özlü, 2017*** | low | low | moderate | low | low | serious | serious | critical |
| ***Abolfazl Rahimi, 2022*** | low | low | moderate | low | low | serious | low | critical |
| ***Shu-Yen Li, 2011*** | low | low | moderate | low | low | serious | serious | critical |
| ***Ayyüce Tuba Koçak, 2021*** | serious | low | low | low | low | moderate | low | serious |

0

.1

.2

.3

.4

.5

Standard error

-2

0

2

4

Hedges's g

**Supplementary Fig.1 Funnel plot of sleep quality,** showing asymmetry with several studies deviating from the expected triangular distribution.

**Supplementary Table S7 Results of global and local inconsistency tests.**

| **global inconsistency test** |  |  |  |  |  |  |  |
| --- | --- | --- | --- | --- | --- | --- | --- |
|  | coefficient | SE | p value | 95%CI | |  |  |
| control–valerian acupressure–acupressure | -0.155 | 1.693 | 0.927 | -3.474 | 3.164 |  |  |
| control–earplug–eyemask–earplug and eye mask | 1.457 | 1.691 | 0.389 | -1.857 | 4.770 |  |  |
| control–eye mask | -1.234 | 1.476 | 0.403 | -4.128 | 1.659 |  |  |
| control–earplug and eye mask | -0.347 | 1.289 | 0.788 | -2.872 | 2.179 |  |  |
| earplug and eye mask–massage | 2.956 | 2.086 | 0.156 | -1.132 | 7.044 |  |  |
|  |  |  |  |  |  |  |  |
| **local inconsistency test** |  |  |  |  |  |  |  |
|  | Direct | | Indirect | | Difference | | |
|  | coefficient | SE | coefficient | SE | coefficient | SE | p value |
| control–earplug | 0.882 | 0.827 | 0.030 | 1.881 | 0.853 | 2.058 | 0.679 |
| control–acupressure | 1.185 | 1.182 | 1.495 | 3.109 | -0.310 | 3.327 | 0.926 |
| control–eye mask | 1.388 | 0.680 | 0.611 | 1.959 | 0.777 | 2.076 | 0.708 |
| control–earplug and eye mask | 1.298 | 0.387 | -1.240 | 1.213 | 2.538 | 1.274 | 0.046 |
| control–massage | -0.988 | 1.108 | 1.578 | 1.195 | -2.566 | 1.629 | 0.115 |
| valerian acupressure–acupressure | 0.308 | 1.178 | -0.002 | 3.113 | 0.310 | 3.327 | 0.926 |
| earplug–eye mask | 0.589 | 1.179 | 0.520 | 1.402 | 0.069 | 1.833 | 0.970 |
| earplug–earplug and eye mask | -0.020 | 1.174 | 0.633 | 1.121 | -0.653 | 1.623 | 0.687 |
| ergonomic sleep masks–earplug and eye mask | -0.378 | 1.135 | 2.128 | 631.805 | -2.506 | 631.806 | 0.997 |
| eye mask–earplug and eye mask | -0.609 | 1.174 | -0.019 | 0.906 | -0.590 | 1.483 | 0.690 |
| earplug and eye mask–massage | 0.366 | 1.129 | -2.200 | 1.175 | 2.566 | 1.629 | 0.115 |

Abbreviations: SE, standard error; CI, confidence interval
